# Atlantic Bottlenose Dolphins (*Tursiops truncatus*) as A Sentinel for Exposure to Mercury in Humans: Closing the Loop

**DOI:** 10.3390/vetsci2040407

**Published:** 2015-11-12

**Authors:** John S. Reif, Adam M. Schaefer, Gregory D. Bossart

**Affiliations:** 1 Department of Environmental and Radiological Health Sciences, Colorado State University, Fort Collins, CO 80523, USA; 2 Harbor Branch Oceanographic Institute at Florida Atlantic University, Fort Pierce, FL 34946, USA; E-Mail: aschaef3@fau.edu; 3 Georgia Aquarium, Atlanta, GA 30313, USA; E-Mail: gbossart@georgiaaquarium.org

**Keywords:** animal sentinels, bottlenose dolphins, epidemiology, fish, marine mammals, mercury, one health

## Abstract

Mercury (Hg) is a ubiquitous global contaminant with important public health implications. Mercury is released from a variety of anthropogenic, industrial processes, enters the earth's atmosphere and is re-deposited onto the earth’s surface in rainfall. Much of this Hg enters the oceans which cover the majority of the earth’s surface. In the marine environment, inorganic Hg is converted to the most toxic form of the element, methylmercury, and biomagnified through the trophic levels of the food web. The bottlenose dolphin (*Tursiops truncatus)* is the apex predator in many estuarine and coastal ecosystems. Due to their long life span and trophic position, bottlenose dolphins bioaccumulate high concentrations of contaminants including Hg, thus making them an important sentinel species for ecosystem and public health. Bottlenose dolphins in Florida bioaccumulate high concentrations of Hg in their blood, skin and internal organs. The concentrations of Hg in blood and skin of bottlenose dolphins of the Indian River Lagoon, FL (IRL) are among the highest reported world-wide. In previous studies, we demonstrated associations between concentrations of total Hg in the blood and skin of IRL dolphins and markers of endocrine, renal, hepatic, hematologic and immune system dysfunction. The predominant manifestation of exposure to mercury in humans is neurotoxicity. During the 1950s and 1960s, residents of Minamata bay, Japan were exposed to high concentrations of methyl mercury as the result of ingestion of fish and shellfish that had become contaminated in this infamous environmental disaster. Affected adults had severe motor and sensory abnormalities often leading to death. Methyl mercury crosses the placenta during pregnancy. Children exposed *in utero* were born with multiple congenital anomalies and also suffered from neurologic disorders. Significantly, local cats that consumed Hg contaminated fish developed severe signs of neurotoxicity which led to their subsequent description as the “dancing cats of Minamata bay”. Unfortunately, the cause of these strange manifestations in cats was not recognized in time to prevent hundreds of additional cases from occurring. More recent studies have shown that exposure to mercury as a result of seafood consumption during pregnancy may result in multiple cognitive and neurodevelopmental effects in children. The levels of mercury found in bottlenose dolphins and the health effects we identified alerted us to the possibility of an important public health hazard. The IRL occupies 40 percent of the east coast of Florida and is bordered by counties with approximately 2.5 million human inhabitants. Therefore, we hypothesized that local inhabitants in communities bordering the IRL could be at risk of exposure to Hg from the consumption of fish and shellfish. We measured hair Hg in 135 local residents and found a mean concentration of 1.53 µg/g which was higher than that from previous studies of sport fishermen and coastal residents in other states. Over 50% of participants had a hair Hg concentration which exceeded the U.S. EPA exposure guideline. Hair Hg concentration was directly related to the frequency of seafood consumption and to the proportion of fish and shellfish obtained from local recreational sources. This study clearly exemplifies the importance of an animal sentinel in identifying a public health hazard and is virtually unique in “closing the loop” between animal and human health.

## 1. Introduction

Mercury (Hg) is a ubiquitous global contaminant with important public health implications. Elemental and particulate-bound ionic forms of mercury are released into the environment from multiple industrial processes such as metal production, waste incineration, mining and the burning of coal for energy as well as natural phenomena such as volcanic eruptions [1]. Inorganic Hg enters the atmosphere, is transported globally and is re-deposited onto the earth’s surface in rainfall and by dry deposition [2]. Much of this re-deposited Hg enters the marine environment, primarily in the oceans, which cover 71 percent of the earth’s surface. Inorganic Hg is converted to methylmercury (MeHg) in aquatic sediments by the action of multiple anaerobic species of sulfate-reducing bacteria [3]. Thus the sulfate concentration and composition of bacterial communities in marine sediments are important predictors of mercury methylation rates [4]. Methylmercury, in turn, is taken up from sediments by phytoplankton and biomagnified through fish species within food webs, reaching the highest levels in the apex predators within an ecosystem. Estuarine ecosystems, including the Everglades and much of southern Florida, are hydro-geological sinks for Hg due to their biogeochemistry, concentrations of sulfates in sediments, pH and patterns of rainfall deposition [5].

## 2. Mercury Bioaccumulation in Dolphins

The Atlantic bottlenose dolphin (*Tursiops truncatus*) is an apex predator in many marine ecosystems. They inhabit estuarine, coastal, and offshore waters along the Atlantic coast of the United States while related species e.g., the striped dolphin (*Stenella* *coeruleoalba)* occupy the same ecologic niche in other coastal waters such as the Mediterranean Sea. Due to their long life span and trophic position as apex predators, bottlenose dolphins bioaccumulate high concentrations of organic and inorganic contaminants including Hg, thus making them an important sentinel species for ecosystem and public health [6,7,8].

It has long been recognized that deceased, stranded bottlenose dolphins from U.S. coastal waters contain high levels of Hg in skin, liver, kidney and muscle [9,10,11,12,13]. Similar observations have been made in coastal dolphins of various species in other hemispheres [14,15,16,17]. However, relatively few studies have measured Hg in healthy, free-ranging individuals. The exceptions are from capture-release health assessments conducted on the east and west coasts of Florida [18,19,20,21]. These studies were conducted to establish baseline values for a suite of trace elements in populations from the Indian River Lagoon (IRL) and Sarasota Bay, Florida. At both sites, high levels of total mercury (THg) were reported in blood and skin obtained at the time of examination (Table 1). Dolphins captured between 2003 and 2005 in the IRL, a 165 mile estuary along Florida’s east-central coast, had mean concentrations of THg of 658 ± 519 μg/L wet weight in blood [18], and 7.0 ± 5.9 μg/g dry weight in skin [19]. These concentrations were more than four times higher than those found in a comparison population of bottlenose dolphins sampled in Charleston harbor, SC during the same time frame. Similar findings were reported in bottlenose dolphins from the west coast of Florida where the concentration of THg in blood was 570 ± 434 μg/L [20]. Approximately 73 percent of THg in the skin of dolphins from the IRL was MeHg [19], while in Sarasota Bay dolphins 96% of the THg in blood was reported to be MeHg [20].

**Table 1:** Reported concentrations of total mercury in blood and skin from bottlenose dolphins.

Study Site	Years	Total Mercury Concentration Mean ± SD		Reference
		Blood (μg/L ± SD)	Skin (μg/g ± SD)	
Indian River Lagoon, FL	2003–2005	658 ± 519.0 (*n* = 75)	7.0 ± 5.9 (*n* = 75)	[18,19]
Charleston, SC	2003–2005	147 ± 88.0 (*n* = 74)	1.7 ± 0.9 (*n* = 74)	[18,19]
Sarasota Bay, FL	2003–2005	570.3 ± 433.5 (*n* = 55)	2.1 ± 1.7 (*n* = 54)	[20]
Sarasota Bay, FL	2002–2004	512 ± 363 (*n* = 51)	2.13 ± 1.54 (*n* = 40)	[21]
Italy, Aquaria	2001	139 ± 220 (*n* = 4)	-	[22]
National Aquarium, Baltimore, MD	2011	63.9 ± 34.0 (*n* = 7)	-	[23]

Subsequently, mean concentrations of THg in the liver of stranded IRL dolphins were reported to be 10 fold higher than those found in stranded dolphins from South Carolina between 2000 and 2008 [13]. Further, a highly significant correlation was found between skin and liver concentrations of THg in stranded dolphins from the IRL, documenting the usefulness of skin, a relatively accessible tissue in live animals [13]. The concentration of THg found in wild Florida dolphins was approximately one order of magnitude greater than that found in dolphins in aquaria in Maryland and Italy, where the concentration of THg in the fish diets were correspondingly lower [22,23]. The levels of THg in blood and skin of Florida bottlenose dolphins are among the highest recorded in free-living marine mammals worldwide. Collectively, these studies demonstrate that dolphins bioaccumulate high concentrations of Hg in a variety of tissues as a result of biomagnification through trophic levels of the food chain.

## 3. The Indian River Lagoon, Florida

The Indian River Lagoon is a biodiverse estuary that occupies approximately 40% percent of the east coast of Florida, spanning a distance of more than 250 km. The estuary has a narrow, linear shape which dictates that dolphins essentially move along a north-south axis [24]. Bottlenose dolphins in the IRL maintain strong site fidelity and have limited home ranges as shown by longitudinal photo-identification studies of individual animals [25]. The estuary contains three major basins, and can be divided into six segments based on unique hydrodynamic and geographic features. The IRL is connected to the Atlantic Ocean by five inlets and one man-made canal, resulting in low rates of water turnover. The shallow depth and the limited tidal exchange between the lagoon and the Atlantic Ocean results in minimal flushing and concentration of microbial and chemical agents in the ecosystem [24].

We recently demonstrated a north to south gradient in THg blood concentrations in bottlenose dolphins of the IRL by incorporating photo-identification data to assign individuals to geographic segments [24]. Intense residential and urban population development and agricultural activity along Florida’s east coast have resulted in increased freshwater inputs and altered water quality leading to decreased seagrass habitat and eutrophication [26,27]. The northernmost areas of the IRL are relatively pristine, while the southern segments are characterized by increased coastal development, greater human population density and a higher concentration of drainage canals from inland areas [24]. These anthropogenic activities have impacted alkalinity, pH, and dissolved organic carbon concentrations which can alter sulfate levels in sediments [28]. Elevated sulfate concentrations in the Florida Everglades sediments increase the conversion rates of inorganic Hg to the methylated form which is bioaccumulated in fish [29].

We also examined temporal trends in THg concentrations in blood of IRL dolphins [24]. From 2003 to 2012, the concentrations decreased significantly over time, consistent with decreases in THg in fish species in the U.S. [30] and in striped dolphins from the Mediterranean Sea [16]. However, although the linear trend in blood THg showed a significant decline between 2003 and 2012, most of the decline was attributable to a high concentration in 2003 (1012 μg/L wet weight), which was between 20 and 58 percent lower and relatively stable between 2004 and 2012 [24]. The decline in Hg concentrations in fish is attributed to reduction of exposure from point sources and emissions. However, trends in fish in the southeastern United States between 1988 and 2005 (not including Florida) did not show the decline found elsewhere in the country [30]. Deposition patterns show increases in this region and may be due to a greater influence of global atmospheric Hg emissions, rather than local sources [30]. This appears to be the case for the IRL, since there are very few point sources of industrial activity along the border of the lagoon. Rainfall deposition data also support this conclusion since over 50% of Hg in south Florida is due to long range global transport of gaseous Hg and deposition rates remain stable [31].

## 4. Health Effects of Exposure to Mercury in Dolphins

Given the evidence that dolphins bioaccumulate high concentrations of Hg in a variety of tissues, we explored the potential that this exposure may result in adverse patho-physiological effects on a variety of organ systems. The study population consisted of 56 bottlenose dolphins from the IRL and 65 from Charleston Harbor, SC captured between 2003 and 2005 during the Bottlenose Dolphin Health and Risk Assessment (HERA) project, a multidisciplinary collaborative effort designed to evaluate the effects of environmental exposures on individual and population health [32]. We evaluated associations between THg and selenium concentrations in blood and skin and endocrine, hepatic, renal and hematological parameters using multiple linear regression models with adjustment for age in a pooled analysis [33]. Selenium plays a protective role in mitigating the toxic effects of Hg through a variety of mechanisms one of which is the formation of mercury-selenium complexes [34]. Recent research demonstrates that selenium may operate through multiple mechanisms including competition with Hg for biological targets, mobilization of mercury stores between organ systems and interactions in the liver and kidney where MeHg is transformed into inorganic Hg [35,36]. Blood THg concentrations were previously reported to be correlated with Se concentrations in IRL dolphins [18].

Increases in blood and skin THg concentrations were associated with a decrease in total thyroxine (T4) and triiodothyronine (T3), demonstrating an effect on endocrine function [32]. In contrast, adrenocorticotropic hormone (ACTH) increased with increasing concentrations of Hg in the blood and skin. No statistically significant associations were found for free T4, testosterone, aldosterone, cortisol, estradiol or progesterone. An effect on liver function was suggested by an increase in gamma-glutamyl transferase (GGT) with increasing THg concentrations in blood and skin; lactic dehydrogenase was positively correlated with skin THg. An effect on renal function was also found with an increase in blood urea nitrogen (BUN) and increasing concentrations of THg in blood and skin; however, no association was demonstrated for creatinine. With respect to hematological parameters, increases in THg in blood and skin were associated with decreases in the absolute numbers of lymphocytes, eosinophils and platelets and an increase in the absolute number of segmented neutrophils [33].

These results were consistent with previous reports. Associations between blood or skin Hg concentrations and thyroid hormones, liver enzymes and several hematologic parameters were described in a study of dolphins from Sarasota Bay, FL [20]. Pathologic changes in the liver consisting of periportal lipofuchsin deposits, fatty infiltration, central lobular necrosis and lymphocytic infiltration were described in dolphins from the west coast of Florida with high concentrations of Hg in liver [37]. In summary, the results suggest the potential for a deleterious effect of Hg on the endocrine, hepatic, renal and hematopoietic systems in highly exposed bottlenose dolphins.

We also studied the effects of increased exposure to Hg on the immune system of bottlenose dolphins and found associations with both innate and acquired immunity, as well as with effects on immune cell populations and immune globulins [38]. Data from 142 dolphins collected during the HERA project from the IRL and Charleston Harbor, SC were analyzed by dividing Hg concentrations in blood and skin into tertiles in a pooled analysis, adjusted for age. Total globulins increased significantly across tertiles of blood Hg concentration due primarily to an increase in gamma globulin. Two measures of innate immunity, monocyte phagocytic activity and plasma lysozyme concentration, increased significantly with increasing concentration of blood Hg. Lymphocyte CD19 + (immature) and CD21 + (mature) B cell markers were reduced significantly. Non-significant reductions in CD2 + and CD4 + helper T cell subpopulations were also observed. Antigen presenting cells expressing MHC class II molecules decreased significantly with increasing categories of Hg exposure. B and T cell lymphocyte proliferation were reduced in a stepwise, dose-dependent manner. Reductions in antibody concentration to common marine micro-organisms suggested that dolphins with high exposures to Hg may be more susceptible to infectious diseases [38]. Increased mortality from infectious disease associated with Hg exposure was also reported in a study of cetaceans from the United Kingdom [39]. Mean liver concentrations of mercury, selenium and zinc were significantly higher in harbor porpoises (*Phocoena phocoena)* that died of infectious diseases compared to healthy porpoises that died from trauma [39].

Mercury is also known to cause immune system effects in humans [40]. Occupational exposures to inorganic Hg were associated with alterations in B lymphocytes, T-helper cells, T-suppressor cells, and T-cell proliferation. These findings led to the hypothesis that exposure to Hg increases risk for infectious diseases in humans, subsequently supported by epidemiologic studies and laboratory based research. Correlations between exposure to Hg and a self-reported history of malaria infections were reported in Amazonian communities exposed through fish consumption in an area of alluvial gold mining activity [41]. These populations also had changes in the serum concentration of antinuclear antibodies (ANA) and antinucleolar antibodies (ANoA), biomarkers for autoimmune disease [42,43] and in their profiles of pro- and anti- inflammatory cytokines [43]. Mercury has also been shown to induce an autoimmune disease resembling lupus erythematosus in rodent models with increases in immunoglobulins IgG and IgE [44], compatible with electrophorectic patterns in exposed dolphins.

## 5. Minamata Disease and Neurotoxicity of Methylmercury in Humans

A variety of health effects have been described in humans following exposure to organic and inorganic Hg [40]. Principal among these is neurotoxicity, initially recognized through two well-documented episodes. Severe neurologic impairment induced by the consumption of fish and shellfish containing high levels of MeHg at Minamata Bay in Japan [45] was the initial harbinger of toxicity. In this well chronicled environmental disaster, waste water discharges containing MeHg, produced as a byproduct from the manufacture of acetaldehyde, were released from a chemical plant during the 1950s and 60s. These discharges led to widespread contamination of the watershed, with high levels of organic Hg in sediments, shellfish and fish [46]. Exposures to adults from consumption of seafood resulted in a syndrome known as “Minamata Disease” characterized by symptoms of severe neurotoxicity; lack of motor coordination, ataxia, muscle weakness, numbness, sensory disturbances, visual and hearing deficits, seizures and death [45]. The ingestion of highly contaminated seafood by pregnant women was followed by an epidemic of severe fetal abnormalities and neurotoxicity in their offspring. Methylmercury crosses the placenta and the blood brain barrier, resulting in high levels in the developing fetus [47]. These children exhibited congenital abnormalities such as microcephaly, and were born with various central nervous system symptoms including blindness, seizures and severe mental and physical developmental retardation [45]. A second large outbreak of neurotoxicity occurred in Iraq in 1971–1972 as the result of consumption of bread made from methylmercury treated grain [48].

Subsequently, more subtle neurodevelopmental effects have been demonstrated in cohort studies of populations which rely heavily on the consumption of fish, shellfish and marine mammals as dietary staples. Developmental neurotoxicity in children exposed prenatally was shown in cohort studies of Faroese women who consumed a high proportion of their diet as seafood including meat from pilot whales [49]. In a second large cohort study conducted in the Seychelles, the results were less clear since the initial reports showed no association between maternal hair mercury and neurodevelopmental test performance [50]. However, more recently, mercury was shown to have a negative effect in this population when maternal fish consumption was taken into account in the analysis [51]. Similarly, in a smaller U.S. cohort, maternal fish consumption was shown to improve performance on tests of cognition in 6 months old children, while performance decreased with increased hair mercury concentrations [52]. In general, low level exposure to mercury during pregnancy is associated with decreased performance on tests of cognitive development and psychomotor performance in children exposed *in utero* but the relationships are complex because of the beneficial effects of maternal fish consumption [53,54]. Prenatal exposure can result in multiple cognitive and neurodevelopmental effects including deficits in memory, language, attention, visual acuity and fine motor skills [52,55]. The prenatal exposure of children to MeHg derived from fish and seafood with resulting impaired neurodevelopment is an important public health concern of international significance [40,56].

## 6. The Dancing Cats of Minamata Bay

The environmental disaster at Minamata bay included an animal sentinel, “the Dancing Cats of Minamata Bay”. Not since the iconic description of “The Canary in the Coal Mine”, has the concept of an early warning system for a human tragedy been so dramatically illustrated. Unfortunately, unlike the miners who heeded the signs of carbon monoxide poisoning and went to the surface, the residents of Minamata were unaware of the significance of events in the local cat population for at least six years [45]. Although the effects of Hg intoxication were amply demonstrated in the animals of the area, nothing was done to prevent or stop the human epidemic.

Harada [45] provides a vivid description. “During the 1950s, people began to witness strange phenomena in and around Minamata Bay. For no apparent reason, fish rotated continuously and floated belly-up to the surface, shellfish opened and decomposed, and birds fell while in flight. The most shocking of all incidents was the frenzied death of cats. Cats suffered from excessive salivation and manifested general convulsions or violent rotational movements, were unable to walk straight, and often collapsed dead. Many jumped into the sea to drown, and eventually cats were no longer seen in the area.” In retrospect, the explanation for the ataxic cats accompanied by convulsions and death became clear. Local cats were fed fish and scraps of fish from the dinner tables of the residents of Minamata [46]. Cats also provided confirmatory evidence of the role of MeHg in the etiology of Minamata disease when laboratory analyses were later performed at Kumamoto University (Table 2) [57]. High concentrations of THg in liver, kidney, brain and hair were found in cats from Minamata compared to tissues from control cats obtained from a fishing village where no cases of Minamata disease had occurred [46,57].

**Table 2:** Concentrations of total mercury (ppm) in fish and shellfish and tissues from cats and humans from Minamata Bay, Japan 1962. (Adapted from [57])

Fish and Shellfish		Cats		Humans	
Oyster	5.6	Control	0.9–3.66	Control	<3.0
Gray Mullet	10.6	Kidney	12.2–36.1	Kidney	3.1–144.0
Clam	20.0	Liver	37–145.5	Liver	0.3–70.5
China Fish	24.1	Brain	8–18.6	Brain	0.1–24.8
Crab	35.7	Hair	21–70	Hair	96–705

A similar incident, albeit of much smaller proportions, occurred in Ontario, Canada in the 1960s when a chloralkali plant released mercury into the river resulting in contamination of fish with MeHg. At least one cat that consumed fish from the river developed a neurological disorder and toxicological analysis of tissues from the cat confirmed Hg as the cause of the illness. Native Americans living in the vicinity who consumed fish had high levels of mercury in hair samples [58].

## 7. The Bottlenose Dolphin as a Sentinel for Human Exposure

As described above, the Atlantic bottlenose dolphin is an apex predator in many marine ecosystems. Bottlenose dolphins in the IRL are long-lived, bioaccumulate high concentrations of organic and inorganic contaminants in blubber and other tissues and exhibit a relatively high degree of site fidelity, making them an excellent sentinel animal for the potential health effects of environmental contamination [8].

Human exposure to MeHg is primarily from the consumption of fish and shellfish [40]. While the general population of the U.S. consumes most of its fish from commercial sources with resulting relatively low levels of Hg in their tissues [59], sport fishermen and women appear to be a high risk group for accumulation of MeHg. Studies of recreational fishermen in several states [60,61,62] have demonstrated Hg concentrations in hair or blood above national averages and higher concentrations among those who ate locally caught sport fish more frequently. Coastal subpopulations often consume more fish and have higher concentrations of Hg in their tissues than the general population [63]. In Florida, the average adult consumes approximately 46 g per day of seafood, considerably higher than the estimated 4.5 g per day for the general population in the United States [64].

The high concentrations of THg found in the blood and skin of bottlenose dolphins in the IRL alerted us to the possibility of exposure to humans in the same area. The lessons learned at Minamata Bay prompted us to undertake a study of Hg exposure among local residents along the IRL [65]. The IRL is bordered by Brevard, Indian River, St. Lucie Martin and Palm Beach counties with a total population of approximately 2.5 million full time residents. Therefore, we hypothesized that a substantial public health problem might exist if the residents of these counties consumed locally caught seafood from the IRL.

We measured exposure to Hg among coastal residents living near the IRL to examine associations between the frequency, species and sources of seafood consumed by residents and their hair Hg concentration [65]. We enrolled residents of coastal counties bordering the IRL using an opportunistic sampling design. Participants provided demographic information and were queried regarding their consumption of fish and shellfish, the frequency and sources of their seafood consumption and the frequency of consumption of 12 common sportfish. Participants provided a hair sample for the determination of THg. Hair has been validated as a reliable biomarker of Hg intake [66] and represents total intake over the prior three months, the same period used in the food frequency questionnaire.

The mean THg concentration in hair for 135 residents was 1.53 µg/g. (Table 3) [65]. The U.S. EPA exposure guideline, which equates to a hair Hg concentration of 1.0 µg/g [67], was exceeded in 50% of participants. The hair Hg concentration among males (2.02 µg/g) was significantly higher than that for females (0.96 µg/g). However, the concentrations in women of all ages in this study were approximately five times higher than those from a randomly generated sample of U.S. women of childbearing age [68]. In addition, the concentrations of hair Hg in this study were higher than those reported from anglers and coastal resident populations in Wisconsin (0.86 µg/g), Louisiana (1.1 µg/g) Alabama (0.55 µg/g) and Canada (0.82 µg/g) [61,62,69,70].

**Table 3:** Concentrations of total mercury in hair (μg/g) among Florida residents, 2011–2012 by demographic and fish consumption variables (Adapted from [65]).

Participant Group	*n*	Mean ± SD	Median	Percentile			*p*-Value
				75th	90th	95th	
All Participants	135	1.53 ± 1.89	1.01	1.86	3.16	5.01	
Sex							<0.01
Male	73	2.02 ± 2.38	1.17	2.81	4.74	6.06	
Female	62	0.96 ± 0.74	0.74	1.38	1.98	2.60	
Total Seafood Consumption							<0.01
≥Once per day	9	2.14 ± 1.86	2.96	3.21	-	-	
Three times per week	66	1.95 ± 2.32	1.20	2.39	4.30	5.30	
Once per week	50	1.08 ± 1.16	0.73	1.41	2.02	2.84	
≤Once per month	10	0.49 ± 0.29	0.39	0.79	0.90	-	
IRL Seafood Consumption							0.11
≥Three times per week	8	2.01 ± 1.47	1.19	3.02	-	-	
Once per week	17	1.71 ± 1.41	1.14	3.07	3.69	-	
≤Once per month	110	1.47 ± 1.99	0.89	1.73	2.96	4.71	
Fish Sources							<0.01
All locally caught	28	2.53 ± 3.20	1.21	3.14	5.36	12.3	
Most locally caught	17	2.46 ± 2.24	1.62	3.76	5.95	-	
Half locally caught	13	1.65 ± 1.06	1.15	2.71	3.44	-	
Most bought from store or restaurant	24	1.20 ± 0.71	1.14	1.73	2.31	2.52	
All bought from store or restaurant	52	0.85 ± 0.73	0.60	1.11	1.85	2.72	
Shellfish Sources							<0.01
All locally caught	14	3.37 ± 4.50	1.20	5.63	12.14	-	
Most locally caught	10	2.54 ± 1.83	1.84	4.47	5.38	-	
Half locally caught	5	2.77 ± 1.15	2.72	3.68	-	-	
Most bought from store or restaurant	19	1.10 ± 0.82	0.85	1.41	2.28	-	
All bought from store or restaurant	85	1.12 ± 1.00	0.75	1.60	2.74	3.08	

Hair Hg concentration was significantly associated with the frequency of total seafood consumption. Individuals who reported consuming seafood once a day or more were almost four times more likely to have a hair Hg concentration over the U.S. EPA reference dose, compared to those who consumed seafood once a week or less. Hair Hg concentration was also significantly higher among individuals who obtained all or most of their seafood from local recreational sources [65].

Concentrations were highest among individuals who reported consuming seafood daily (2.14 µg/g) and decreased with lower categories of total consumption (Table 3). Individuals who consumed seafood from the IRL three times a week or more and those who reported that all fish and shellfish consumed were from local recreational sources had the highest hair Hg concentrations (2.01 µg/g and 2.53 µg/g, respectively). Total hair Hg concentrations were significantly higher among individuals who consumed snapper, sea trout, cobia, and grouper once a week or more compared to those who ate these fish less than once a week [65]. A direct comparison of Hg contamination in fish consumed by dolphins and humans was not undertaken since IRL dolphins consume a majority of their diet as striped mullet, silver perch and spot [71].

## 8. Conclusions

Our recent study of coastal residents in Florida [65] marks the first investigation to apply findings from bottlenose dolphins as a stimulus to explore the potential for similar risk among humans from the same geographical region. Thus, by applying the knowledge gained from the study of this marine mammal sentinel, we have “closed the loop” between animal and human health in a virtually unique manner. In the general area of animal sentinels for human disease of non-infectious origin, very few have linked the findings in the sentinel to a corresponding human exposure or health effect. One exception is the seminal study of mesothelioma in the dog [72] in which asbestos exposure in the dog was linked to occupational exposure or hobbies among the owners in most of the cases.

Following publication of the paper describing Hg exposure in humans in Florida [65], the topic received considerable attention in the local media. There were more than 10 news stories on television, radio and in local newspapers. These publications helped to raise awareness of potential risks of consuming locally caught sportfish and shellfish, especially by pregnant women. Thus, we assume the animal sentinel had a positive effect on public health through risk communication with members of the local population. In a study conducted in Wisconsin, more than two thirds of the women who consumed sportfish were not aware that fish consumption guidelines had been issued to protect against MeHg [63]. We are currently evaluating knowledge, attitudes and practices regarding the hazards of fish consumption and exposure to Hg among pregnant women in Florida. This issue is timely in view of the fact that fish consumption is widely recommended as a means of reducing the risk of cardiovascular disease due to the high levels of n-3 polyunsaturated fatty acids present in the tissues of many species [73].

The current attention being devoted to Oceans and Human Health internationally [74] suggest that additional studies of marine mammal sentinels should be conducted in a wide range of species and locales. Multiple environmental challenges face the animals of the ocean and the humans who reside in the vicinity or may be impacted indirectly. Animal sentinels may be useful in evaluating risks to humans from climate change [75], harmful algal blooms [76], emerging chemical contaminants [77], oil spills [78] and multiple other contemporary threats to ecosystem and public health.
